# Safety Assessment of a *Nham* Starter Culture *Lactobacillus plantarum* BCC9546 via Whole-genome Analysis

**DOI:** 10.1038/s41598-020-66857-2

**Published:** 2020-06-24

**Authors:** Nipa Chokesajjawatee, Pannita Santiyanont, Kanittha Chantarasakha, Kanokarn Kocharin, Chinae Thammarongtham, Supatcha Lertampaiporn, Tayvich Vorapreeda, Tanawut Srisuk, Thidathip Wongsurawat, Piroon Jenjaroenpun, Intawat Nookaew, Wonnop Visessanguan

**Affiliations:** 1 National Center for Genetic Engineering and Biotechnology (BIOTEC), 113 Thailand Science Park, Phahonyothin Road, Khlong Nueng, Khlong Luang, Pathum Thani 12120 Thailand; 2 Pilot Plant Development and Training Institute, King Mongkut’s University of Technology Thonburi (Bangkhuntien), Bangkok, 10150 Thailand; 3 Department of Biomedical Informatics, College of Medicine, University of Arkansas for Medical Sciences, Little Rock, AR 72205 USA

**Keywords:** Microbiology, Bacterial genes

## Abstract

The safety of microbial cultures utilized for consumption is vital for public health and should be thoroughly assessed. Although general aspects on the safety assessment of microbial cultures have been suggested, no methodological detail nor procedural guideline have been published. Herein, we propose a detailed protocol on microbial strain safety assessment via whole-genome sequence analysis. A starter culture employed in traditional fermented pork production, *nham*, namely *Lactobacillus plantarum* BCC9546, was used as an example. The strain’s whole-genome was sequenced through several next-generation sequencing techniques. Incomplete plasmid information from the PacBio sequencing platform and shorter chromosome size from the hybrid Oxford Nanopore-Illumina platform were noted. The methods for 1) unambiguous species identification using 16S rRNA gene and average nucleotide identity, 2) determination of virulence factors and undesirable genes, 3) determination of antimicrobial resistance properties and their possibility of transfer, and 4) determination of antimicrobial drug production capability of the strain were provided in detail. Applicability of the search tools and limitations of databases were discussed. Finally, a procedural guideline for the safety assessment of microbial strains via whole-genome analysis was proposed.

## Introduction

In recent years, the importance of safety assessment of microorganisms used for human consumption has been widely recognised. The majority of the studies on this topic usually focused on the safety profile of probiotic cultures. In contrast, the safety of starter cultures used in food fermentation has not been as thoroughly examined. Similar to probiotics, many starter cultures are consumed alive in large quantity. Generally, the safety of starter cultures, notably those belonging to the lactic acid bacteria (LAB) group, is presumed from substantial histories of safe consumption of fermented foods. Regardless, cases of LAB infection have been observed in patients with underlying medical conditions^1–3^. Hence, the safety of all new microbial strains introduced into the food chain should be assessed to improve food safety and public health.

In this study, the safety of *Lactobacillus plantarum* BCC9546, a starter culture used for fermentation of *nham* was investigated. *Nham* is a fermented pork product popularly consumed in Thailand and neighbouring countries. *Lactobacillus plantarum* is the main LAB responsible for *nham* fermentation, presenting as the most abundant species in the final fermented product^4^. The starter culture *L. plantarum* BCC9546 was isolated from *nham* in 1999^5^ and commercially available since 2001.

**Figure 1:**
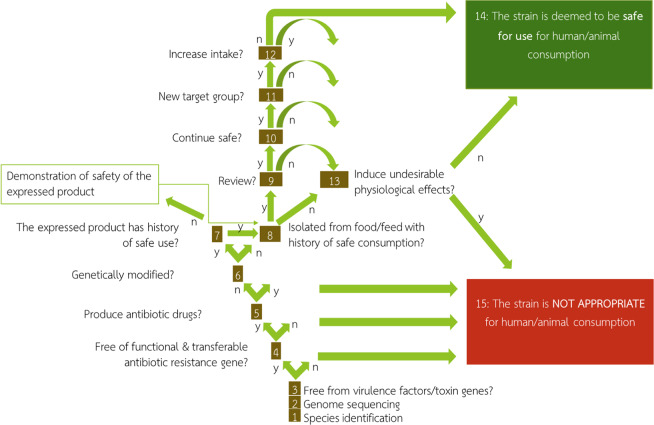
Schematic diagram of a decision tree for safety assessment of microbial cultures to be consumed by humans or animals as proposed by Pariza *et al*.^6^.

With current advances in genome sequencing technologies, safety evaluation of microbial strains can be done at a much higher resolution through whole-genome sequence analysis. The whole-genome analysis has gained increasing attention and was recommended as a part of strain identification and safety evaluation process by Pariza *et al*.^6^. A thirteen-question decision tree proposed by the authors (Fig. 1) was utilized as a guideline for the safety evaluation of starter culture in this study. This study focused on answering the first five questions of the decision tree that can be addressed through the whole-genome analysis. In addition to the safety information of BCC9546, the detailed procedures, resources and precautions in each step were also discussed. Through the application of whole-genome analysis, we propose a procedural guideline for the safety assessment of a microbial strain.

## Results and Discussion

### *L. plantarum* BCC9546 whole-genome sequencing

**Figure 2:**
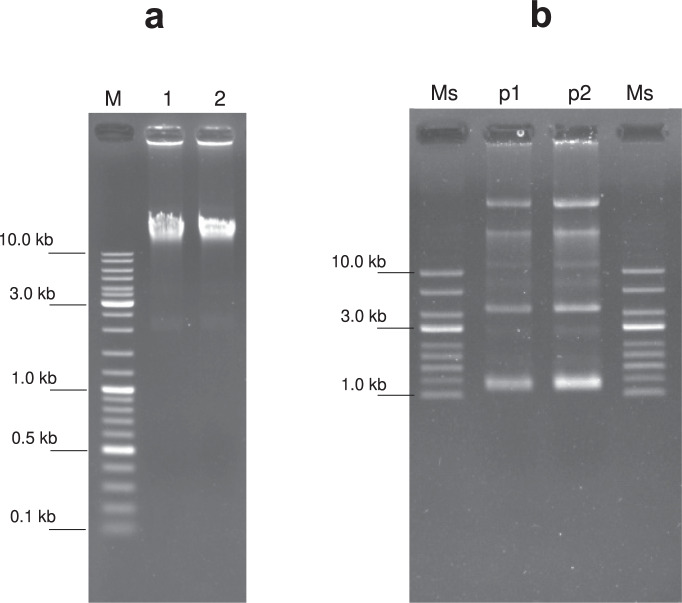
Agarose gel electrophoresis of DNA extracts from BCC9546. (**a**) Genomic DNA running on 1% agarose gel: M, 250 ng DNA size marker (GeneRuler DNA Ladder Mix, Thermo Scientific); 1–2, 250 ng BCC9546 genomic DNA extract. (**b**) Plasmid DNA running on 0.5% agarose gel: Ms, 250 ng supercoiled DNA Ladder (New England BioLabs); p1, 50 ng BCC9546 plasmid DNA extract; p2, 120 ng BCC9546 plasmid DNA extract. The gel images were cropped for concise visualization. See Supplementary Fig. S1 for the uncropped gel images.

High molecular weight genomic DNA and plasmid DNA were successfully extracted from the fresh culture of *L. plantarum* BCC9546. Several bands of plasmid DNA, with the smallest size of approximately 2–3 kb, were visible on the agarose gel (Fig. 2).

The results obtained from the two whole-genome sequencing platforms were shown in Table 1. The sequences obtained from Pacific Biosciences RS II SMRT (PacBio) sequencing platform yielded three unique contigs, i.e., one chromosome and two mega-plasmids. However, this result was deemed insufficient due to the lack of a 2.2 kb plasmid known to be present in this strain (pLpB9, GenBank accession EU391630.1) in the final assembly. This limitation was most likely due to the highly effective size-selection step in the PacBio protocol. The procedure was highly selective for large DNA fragments while excluding smaller plasmid DNAs from the sequencing reaction. Often, the PacBio sequencing was regarded as the platform providing complete genomic information as both chromosome and large plasmids, usually with the size larger than 10 kb, were detected^7,8^. Thus, the presence of small plasmids and their associated risk were not determined.

**Table 1:** The BCC9546 whole-genome sequence obtained from the Pacific Biosciences (PacBio) and the hybrid Oxford Nanopore Technologies and Illumina (ONT-Illumina) platforms.

Contig	PacBio		Hybrid ONT-Illumina	
	contig size (bases)	average read coverage	contig size (bases)	average read coverage
Chromosome	3218570*	372×	3216170	45×
Plasmid A	52070*	172×	52071	72×
Plasmid B	41908*	124×	41908	110×
Plasmid C	—	—	6352*	877×
Plasmid D	—	—	2414*	2314×
Plasmid E	—	—	2271*	3726×

^*^The sequences selected for the safety evaluation of *L. plantarum* BCC9546; - not detected.

In comparison, the hybrid Oxford Nanopore Technologies (ONT)-Illumina sequencing platform yielded six unique contigs. The largest three contigs were similar to those obtained from the PacBio platform. However, this hybrid platform also identified three additional contigs named plasmid C, D, and E. The smallest plasmid E was nearly identical in size and sequence to the known 2.2 kb pLpB9 plasmid. This finding indicated the completeness of the genome sequenced by hybrid ONT-Illumina platform.

Although the hybrid ONT-Illumina platform was shown to be optimal in providing the complete genome information of the strain harbouring small plasmids such as BCC9546, the chromosome obtained from this technique was 2,400 bp shorter than that obtained from the PacBio platform. This reduced chromosome size was due to minor loss of repetitive sequences with length longer than the Illumina reads (>150 bp) during the polishing step in the hybrid assembly. Some repetitive sequences were identified as redundancy of identical chromosomal section, hence wrongfully omitted from the final assembly. Since this assembly error will affect only the copy number of continuous repetitive sequences, it could be considered as a minor flaw and should not affect the overall safety evaluation outcome of the strain.

Therefore, the limitations of the selected sequencing platform should be noted. Completeness of the genome data is crucial for the safety evaluation of a microbial strain. The selected platform should be able to identify all existing plasmids in the genome since plasmids can be the source or result of horizontal gene transfer that may contain virulence factors and/or antimicrobial resistance (AMR) genes. In the case of the PacBio platform, sequencing should be done in a way that small plasmids, if present in the genome, are included in the sequencing reactions, i.e., omitting the size-selection step. However, it should be noted that this action may compromise the average read length and overall efficiency of the long-read sequencing.

**Figure 3:**
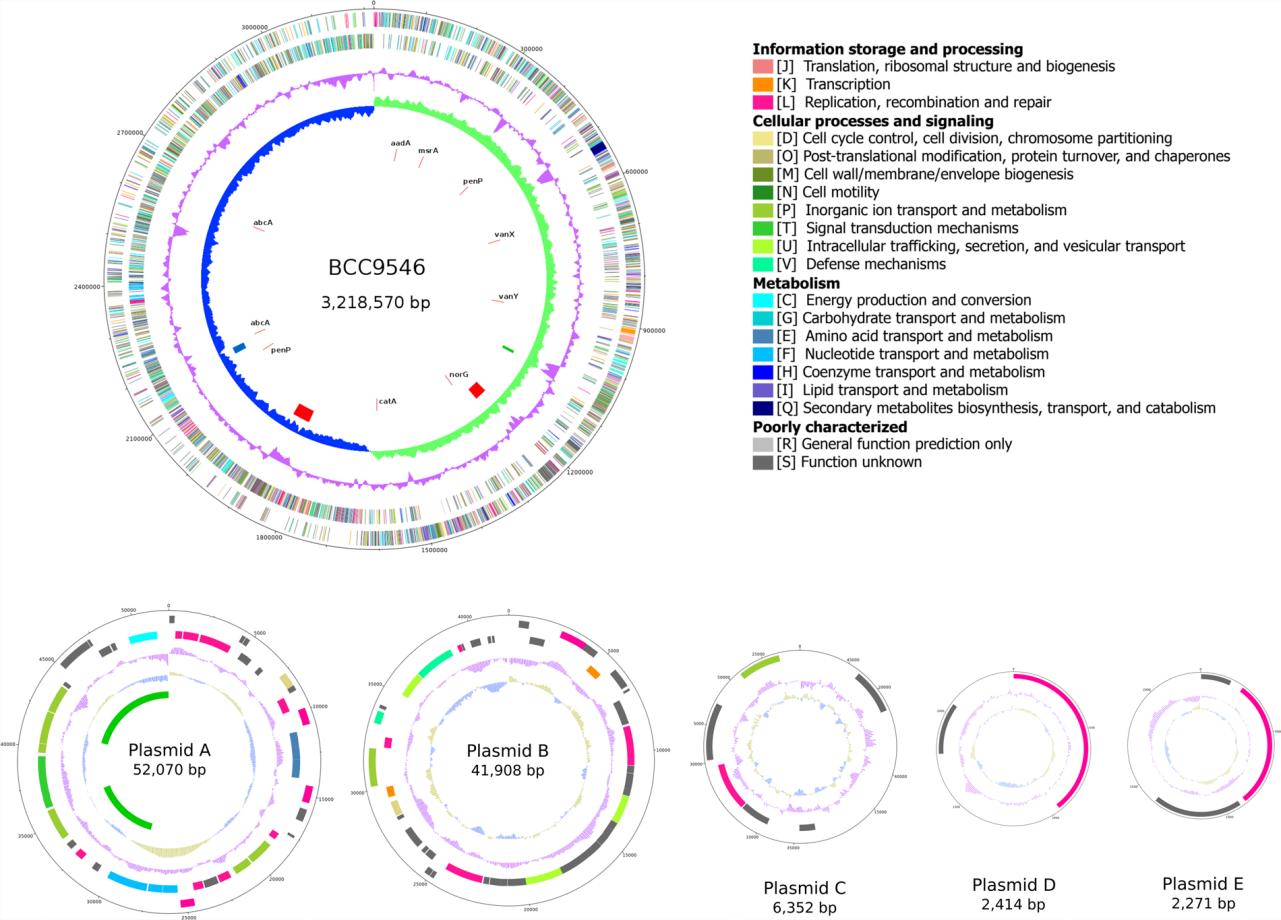
Genome map of *Lactobacillus plantarum* BCC9546. Circles (from outside to inside): circle 1 and 2 are colour-coded according to the COG classification of the genes present on the forward and reverse strands respectively; circle 3 (purple) shows the GC plot; circle 4 (blue and green) represents the GC skew; circle 5 shows incomplete prophage (green), questionable prophage (blue), and intact prophages (red); circle 6 shows antimicrobial resistance genes. The figure is for illustrative purpose only, not drawn to scale.

For the safety evaluation of BCC9546, the chromosome and two plasmids (plasmid A and B) obtained from PacBio and three small plasmids (plasmid C, D, and E) obtained from hybrid ONT-Illumina were selected as the complete genome of this strain. The BCC9546 genome consisted of the main chromosome size 3,218,570 bp with a 44.6% GC content, and five plasmids (A-E) ranging from 52,070 bp to 2,271 bp (Fig. 3). Total of 64 tRNA genes and 16 rRNA genes (Five copies of 16S and 23S rRNA genes and six copies of 5 S rRNA genes) were predicted.

### Species identification

The BCC9546 genome contains five copies of 16S rRNA gene with four sequence variants (≥99.74% identical to one another). The 16 S rRNA gene sequences were highly similar to those of several *Lactobacillus* species, i.e., 99.80–99.93% to *L. plantarum* ATCC 14917 ^T^, 99.87–99.94% to *L. pentosus* DSM 20314 ^T^, 99.68–99.87% to *L. paraplantarum* DSM 10667 ^T^, 98.73–98.93% to *L. xiangfangensis* LMG 26013 ^T^, and 98.89–99.09% to *L. fabifermentans* DSM 21115 ^T^. All of these similarity values were higher than the 98.7% cut-off threshold for species identification as proposed by Chun *et al*.^9^. This result indicated that the analysis using only the 16 S rRNA gene alone could not be used to identify species of the bacteria in this group, especially to distinguish between *L. plantarum* and *L. pentosus*.

The whole-genome sequence was then used to confirm the species of BCC9546. By considering the average nucleotide identity (ANI) value, this strain had the highest ANI value of 98.74% to *L. plantarum* ATCC 14917 ^T^. The ANI values to the other four species were much lower than the 95–96% cut-off threshold as proposed by Richter and Rosselló-Móra^10^, i.e., 85.26%, 79.57%, 75.59%, and 74.68% for the type strain of *L. paraplantarum*, *L. pentosus*, *L. xiangfangensis*, and *L. fabifermentans*, respectively. Therefore, the whole-genome data supported that the strain BCC9546 indeed belongs to the species *L. plantarum*.

The accurate taxonomic placement is crucial for the identification of possible risk associated with the taxon. Following the protocol used in this study, the problem of misidentification or inability to distinguish between closely related species as seen in the identification based on biochemical assays and 16 S rRNA gene analysis^7,11^ can be alleviated.

### Determination of virulence factors and toxin genes

The virulence factor database (VFDB)^12^ was used to identify known virulence factors and toxin genes that may exist in the BCC9546 genome. No virulence genes were found under the stringent criteria of >80% identity and >60% coverage. However, when a set of less stringent criteria (>60% similarity, >60% coverage, and E-value <1e-10) was used, a total of 51 hits were found. Among these hits, hemolysin III (chr_02698) was the only toxin gene identified within the genome. The remaining matches were mostly essential genes for cellular function and adaptation, such as genes involved in cell wall/membrane/envelope biogenesis and attachment (see supplementary file Supplementary_VFDB.xlsx for the complete list of all hits and the manual blast identification of the selected 51 hits). These genes were identified as virulence factors in the virulence factor database as they were also involved in pathogenic bacterial adaptation, survival, or attachment in the hostile/host environment. However, without other pathogenesis mechanisms, these genes could be regarded as beneficial to the bacterium since they increase the bacterial  fitness and may be desirable where live cells are needed (i.e., in the case of starter cultures and probiotics).

For the gene identified as hemolysin, the manual investigation through blastp search confirmed the gene identification with 100% identity to “predicted membrane channel-forming protein YqfA, hemolysin III family” of *Lactobacillus*. Notably, the gene was also observed in several commercial probiotics such as an approved Generally Recognised as Safe (GRAS) probiotic strain *L. plantarum* 299 V, a widely used commercial probiotic in China *L. plantarum* JDM1, a kimchi probiotic *L. plantarum* ST-III, and many other *Lactobacillus* strains in the GenBank database. Hemolysis test using sheep-blood agar showed a hazy zone of hemolysis around the bacterial growth similar to the area around the probiotic strain 299 V, indicated a similar hemolysis activity of the two strains (see Supplementary Fig. S2). Since the hemolysin III gene is widespread in *Lactobacillus* spp. and the strains harbouring the gene have been proven for their safety and are commercially available in many countries, the bacterium harbouring this gene should not be of safety concern, provided that no other pathogenesis genes are present in the genome.

In the search for bacterial toxins using the Kyoto Encyclopedia of Genes and Genomes (KEGG) database^13^, the hemolysin III gene was identified as the only toxin gene in BCC9546 genome. This search strategy facilitated the finding of the relevant toxin genes without a large number of false positives (i.e., the genes involved in adaptation or survival) as seen in the search using VFDB.

In comparison, Zhang *et al*.^14^ identified virulence factors in *L. plantarum* JDM1 using the VFDB database with different stringency criteria, i.e., 70% coverage and 30% identity. The study found as many as 126 hits. However, most of the genes were identified as defensive or non-classical virulence factors, and none were found to be offensive virulence factors. Interestingly, the study failed to identify the hemolysin III gene in the JDM1 genome. This finding raised awareness of the need for consensus criteria or a harmonized protocol for the optimal safety assessment process. In this study, the search using the KEGG database was shown to be accurate and efficient for the purpose.

### Biogenic amine production

The ability to produce biogenic amines (BA) should be assessed for microbial strains intended to be used in food, especially in fermented food where high microbial activities may result in undesirable BA accumulation. As recommended by the European Food Safety Authority (EFSA), the BA-nonproducing starter cultures should be used to control the risk due to BA^15^. A bacterial strain lacking genes involved in BA production can be considered as BA-nonproducing strain and deemed safe for this aspect. In the case that the genes were found, the actual production and accumulated levels of the specific BA at the intended use condition should be determined to quantify the actual risk. To identify the presence of the genes involved in BA production, we found that the KEGG database search was efficient since the enzymes involved in all significant BA-production pathways were included. There were no genes related to the production of BA, including cadaverine, putrescine, spermidine, spermine, ornithine, histamine, tyramine, and tryptamine in the genome of BCC9546. Therefore, this strain can be considered as BA-nonproducer and poses no safety concern in this aspect.

### D-lactic acid production

In the search using the KEGG database, two genes responsible for the production of D-lactic acid, i.e., lactate racemase (chr_00083) and D-lactate dehydrogenase (chr_00684 and chr_01677) were identified. The production of D-lactic acid by the strain was confirmed through HPLC method based on chiral analysis of an 18 h-cultured medium. The strain produced approximately 22 g/L lactic acid, in which approximately half were present in the D-configuration (see Supplementary information). Since D-lactic is an essential component in cell wall peptidoglycan of several gram-positive cocci including *L*. *plantarum*, production of D-lactic could be seen as an intrinsic property of the bacteria in this group. Therefore, a general precaution for the consumption of D-lactic producing bacteria should be provided to those with a high risk of D-lactic acidosis, such as in patients with short-bowel syndrome or carbohydrate malabsorption^16^.

### Bile salt deconjugation

Through KEGG database search, four copies of the gene encoding choloylglycine hydrolase (chr_00054, chr_02114, chr_02755, chr_02880) were found in the BCC9546 genome. The presence of the choloylglycine hydrolase (also known as bile salt hydrolase) gene is an indication of bile salt deconjugation capability. In the *Guidelines for the Evaluation of Probiotics in Food* issued by FAO/WHO, bile salt hydrolase activity was included among desirable properties of probiotics such as resistance to gastric acidity, bile acid resistance, and adherence^17^. However, in the same document, bile salt deconjugation was listed as one of the properties that should be characterised for safety assurance. A comprehensive review by Begley *et al*.^18^ reported several studies regarding the beneficial effects of the bile salt deconjugation activity on the survival of bacteria and the cholesterol-lowering impact on the host. However, a high level of deconjugated bile may compromise normal lipid digestion, disrupt normal intestinal conditions, induce gallstone, and may be further modified to carcinogenic secondary bile salts^18^. After weighing all the benefits and concerns using the scientific evidence shown above, we propose that the bile salt deconjugation property could be seen as desirable when the strain is incapable of modifying the deconjugated biles into the harmful secondary bile products. Aside from the choloylglycine hydrolase, no genes related to the secondary bile salts biosynthesis were found in BCC9546. This concludes the strain’s bile salt deconjugation capability, which may play an essential role in the host digestive system survival. With regards to its inability to produce the harmful secondary bile products, we deem BCC9546 poses no safety concern from this property.

### Antimicrobial resistance (AMR) phenotype

The minimum inhibitory concentrations (MICs) and the microbiological cut-off values of the tested antimicrobials were shown in Table 2. BCC9546 exhibited susceptibility to most antimicrobials tested. However, there were two drugs, i.e., chloramphenicol and kanamycin, in which the MICs were above the cut-off values, indicating acquired resistance for these drugs. Thus, these acquired resistances and their possibility of transfer were further investigated.

**Table 2:** Antimicrobial resistance phenotype of *L. plantarum* BCC9546.

No.	Antimicrobials	Microbiological cut-off ^a^ (mg/L)	MIC^b^ (mg/L)	Interpretation
1	Ampicillin	2	1	Sensitive
2	Chloramphenicol	8	16	Resistance
3	Clindamycin	2	2	Sensitive
4	Erythromycin	1	1	Sensitive
5	Gentamicin	16	16	Sensitive
6	Kanamycin	64	128	Resistance
7	Tetracycline	32	32	Sensitive

^a^Microbiological cut-off values for *Lactobacillus plantarum/pentosus* according to EFSA, 2012^19^

^b^Minimum inhibitory concentrations for *L. plantarum* BCC9546 determined in this study

In this study, we propose the use of a limited number of antimicrobials, i.e., 7–9 depending on the bacterial group, as recommended by the current version of EFSA^19^ over the more extensive lists documented elsewhere^14,20^. The EFSA document was chosen as a guideline due to indication of clear microbiological cut-off values that can be used to distinguish between intrinsic and acquired resistance for bacterial groups commonly used in food and feed. It should be noted that the antimicrobial breakpoints widely reported elsewhere usually refer to clinical breakpoints, not microbiological cut-offs. Since the primary purpose of the clinical breakpoints is to identify the choice of drugs for effective treatment, often for infection by specific pathogens, hence they are usually not determined and less relevant in the case of non-pathogenic bacteria. However, in addition to the minimum list as recommended by EFSA, the resistance to other antimicrobials may be investigated if the cut-off values are known or if it fits the purpose of the study.

### Antimicrobial resistance gene

The search using two AMR databases, CARD^21^ and ResFinder^22^, with the default settings (perfect/strict option for CARD; 90% threshold and 60% minimum length for ResFinder) returned no hits for AMR genes in BCC9546 genome. However, under a less stringent criterion (perfect/strict/loose option in CARD), 273 hits were predicted as AMR genes with ranges from 19–61% identity and 16–307% coverage. Due to the low stringency of the search criterion, the majority of the hits were not actually AMR genes. Nonetheless, we found one gene (Chr_1468) with 100% identity to a gene encoding chloramphenicol acetyltransferase (*cat*) which is responsible for the chloramphenicol resistance in several *Lactobacillus* species. This *Lactobacillus cat* gene possesses 28% best identity match to the *cat* gene of *Enterococcus faecalis* that was included in the CARD database. Inability to identify the *cat* gene in BCC9546 at the default, high stringency setting may be due to the limited repertoire of AMR genes included in the databases. Since both CARD and ResFinder databases mainly focus on AMR determinants of pathogenic bacteria, the AMR genes of non-pathogenic bacteria such as those from *Lactobacillus* are usually not included. Therefore, the limitation of AMR gene search using current version of CARD and ResFinder databases for non-pathogenic bacteria should be noted.

On the contrary, the KEGG database search yielded ten AMR-related genes in the BCC9546 chromosome (Table 3). The *cat* gene was promptly identified. While no specific gene for kanamycin inactivation was found, several genes related to the efflux pumps conferring multidrug resistance were identified in the genome. These efflux pumps may contribute to the kanamycin resistance trait of the strain. Additionally, the presence of *aadA* gene indicated possible streptomycin resistance. Notably, the presence of a macrolide resistance gene *msrA* did not confer resistance to erythromycin, the macrolide antibiotic used in this study. This may be due to several factors such as the gene expression level and the substrate specificity of the expressed product. Similarly, despite the possession of two beta-lactamase genes, the strain was sensitive to ampicillin. Since beta-lactamase is a large enzyme family with variations in their substrate specificity^23^, resistance to other beta-lactam drugs cannot be excluded without further investigation.

**Table 3:** List of antimicrobial resistance genes and their locations in BCC9546 genome.

No.	Resistance	KEGG_ID	Gene Name	BCC9546 gene ID (location)
1	Aminoglycoside resistance	K00984	*aadA* streptomycin 3”-adenylyltransferase [EC:2.7.7.47]	BCC9546_chr_00077 (87890..88669)
2	Macrolide resistance	K18231	*msrA, vmlR* macrolide transport system ATP-binding/permease protein	BCC9546_chr_00175 (196973..198493)
3	Phenicol resistance	K19271	*catA* chloramphenicol O-acetyltransferase type A [EC:2.3.1.28]	BCC9546_chr_01464 (1597918..1598586)
4	beta-Lactam resistance	K17836	*penP* beta-lactamase class A [EC:3.5.2.6]	BCC9546_chr_00384 (407686..408519)
5	beta-Lactam resistance	K17836	*penP* beta-lactamase class A [EC:3.5.2.6]	BCC9546_chr_01967 (2122487..2123617)
6	Vancomycin resistance	K07260	*vanY* zinc D-Ala-D-Ala carboxypeptidase [EC:3.4.17.14]	BCC9546_chr_00808 (896088..896825)
7	Vancomycin resistance	K08641	*vanX* zinc D-Ala-D-Ala dipeptidase [EC:3.4.13.22]	BCC9546_chr_00590 (650889..651446)
8	Multidrug resistance	K18104	*abcA, bmrA* ATP-binding cassette, subfamily B, bacterial AbcA/BmrA [EC:7.6.2.2]	BCC9546_chr_02046 (2192301..2194064)
9	Multidrug resistance	K18104	*abcA, bmrA* ATP-binding cassette, subfamily B, bacterial AbcA/BmrA [EC:7.6.2.2]	BCC9546_chr_02432 (2622491..2624248)
10	Multidrug resistance	K18907	*norG* GntR family transcriptional regulator, regulator for abcA and norABC	BCC9546_chr_01156 (1283935..1285386)

### Mobile genetic elements

The main concern regarding AMR genes in beneficial non-pathogenic bacteria is for their transfer possibility to other possibly pathogenic bacteria which may lead to complications, reducing effectiveness of the antibiotic treatment. To identify this risk, we focused on two types of mobile elements, i.e., plasmids, and bacteriophages, since they are the most likely vehicles involved in inter-cellular genetic exchange through transformation/conjugation and transduction process, respectively. No *oriT* was found in any of the plasmids, indicating that these plasmids are incapable of self-transmission through conjugative transfer. For the presence of bacteriophage, the PHASTER tool^24,25^ identified four prophage regions in the main chromosome and two regions in plasmid A (Table 4 and Fig. 3, see Supplementary_prophage.xlsx for details). None of the AMR genes were located within the prophage regions, nor in any of the plasmids. Therefore, we concluded that the AMR genes present in BCC9546 have low risks of being transferred to other bacteria; hence the strain poses no safety concern regarding the functional and transferrable AMR property.

**Table 4:** List of prophages and their locations in BCC9546 genome.

Region	Prophage size	Completeness of prophage	Total number of genes	Location
**Chromosome**				
1	7.6 Kb	incomplete	7	1048276–1055938
2	39.8 Kb	intact	52	1207270–1247142
3	56.3 Kb	intact	52	1828216–1884590
4	20.9 Kb	questionable	23	2160909–2181830
**Plasmid A**				
1	7.7 Kb	incomplete	8	28195–35911
2	10.7 Kb	incomplete	11	41324–52032

### Antimicrobial drug production

To limit the emergence of new AMR sub-populations, the microbial strains used for consumption should not produce antimicrobial drugs, especially those determined as critically important for medical treatment. For this purpose, the World Health Organization’s List of Critically Important Antimicrobials for human medicine (WHO CIA list)^26^ was used as reference. Based on the pathway search in the current KEGG database, BCC9546 does not possess the ability to produce the antimicrobials of concern; hence poses no safety concern for this aspect.

### Safety conclusion of *L. plantarum* BCC9546

The starter culture *L. plantarum* BCC9546 was shown to be relatively safe with no transferable AMR genes in the genome. As shown in Table 5, all genes related to the virulence, undesirable metabolites, and AMR identified in BCC9546 were also present in other *L. plantarum*, including several probiotic strains 299 V, JDM1, ST-III, and the reference strain WCFS1. These genes seem to be ubiquitously present within the species. Therefore, we concluded that the starter culture *L. plantarum* BCC9546 is safe, at a comparable safety level to the existing probiotic strains.

**Table 5:** Comparison of amino acid sequences of the undesirable genes found in BCC9546 to those found in other *L. plantarum* probiotic strains.

BCC9546 Gene ID	Gene product	299 V (GCA_001888735.1) % identity (% similarity)	JDM1 (GCA_000023085.1) % identity (% similarity)	ST-III (GCA_000148815.2) % identity (% similarity)	WCFS1 (GCA_000203855.3) % identity (% similarity)
chr_02698	HemolysinIII	99.53 (100.00)	99.53 (100.00)	99.53 (100.00)	99.53 (100.00)
chr_00083	Lactate racemase	99.76 (99.76)	99.76 (99.76)	99.76 (99.76)	99.53 (99.53)
chr_00684	D-lactate dehydrogenase	100.00 (100.00)	100.00 (100.00)	100.00 (100.00)	100.00 (100.00)
chr_01677	D-lactate dehydrogenase	100.00 (100.00)	100.00 (100.00)	100.00 (100.00)	100.00 (100.00)
chr_00054	Choloylglycine hydrolase	98.75 (99.06)	100.00 (100.00)	99.06 (99.37)	99.06 (99.37)
chr_02114	Choloylglycine hydrolase	98.42 (98.74)	100.00 (100.00)	98.42 (98.74)	98.11 (98.11)
chr_02755	Choloylglycine hydrolase	100.00 (100.00)	99.39 (99.70)	100.00 (100.00)	100.00 (100.00)
chr_02880	Choloylglycine hydrolase	99.69 (100.00)	100.00 (100.00)	99.69 (100.00)	99.69 (99.69)
chr_00077	AMR: Aminoglycoside resistance	99.23 (99.61)	99.61 (99.61)	99.23 (99.61)	99.23 (99.61)
chr_00384	AMR: beta-Lactam resistance	99.63 (99.63)	98.52 (98.89)	99.63 (99.63)	98.52 (98.89)
chr_01967	AMR: beta-Lactam resistance	99.71 (99.71)	99.71 (99.71)	99.71 (99.71)	99.14 (99.43)
chr_00175	AMR: Macrolide resistance	98.99 (99.39)	98.79 (98.99)	99.19 (99.39)	99.39 (99.39)
chr_01464	AMR: Phenicol resistance	99.10 (99.10)	99.55 (99.55)	99.10 (99.10)	99.10 (99.10)
chr_00808	AMR: Vancomycin resistance	100.00 (100.00)	99.59 (99.59)	99.59 (100.00)	100.00 (100.00)
chr_00590	AMR: Vancomycin resistance	100.00 (100.00)	99.46 (99.46)	100.00 (100.00)	100.00 (100.00)
chr_02046	Multidrug resistance, efflux pump	100.00 (100.00)	99.49 (99.83)	100.00 (100.00)	100.00 (100.00)
chr_02432	Multidrug resistance, efflux pump	99.83 (100.00)	99.66 (99.83)	99.83 (100.00)	99.83 (99.83)
chr_01156	Multidrug resistance, efflux pump	100.00 (100.00)	99.38 (99.59)	99.79 (100.00)	100.00 (100.00)

### Procedural guideline for the safety assessment of a microbial strain via whole-genome analysis

To the best of our knowledge, despite suggestions on the issues that should be investigated for microbial safety assessment^6,17^, no clear procedural guideline have been provided. Despite several studies reported on microbial safety assessements^7,11,14,27^, no consensual procedure agreement can be observed. Therefore, we would like to propose the guideline for conducting the microbial safety evaluation using the whole-genome analysis as follow:

1. **Determination of the smallest extra-chromosomal DNA size**. The smallest size of the plasmid, if present, in the genome should be determined. This information is required to confirm the completeness of the whole-genome sequence obtained from the selected sequencing platform. This step can be achieved through common laboratory technique such as agarose gel electrophoresis of the plasmid DNA extracted from the strain of interest.
2. **Selection of the whole-genome sequencing platform**. The sequencing platform and protocol used should provide complete information of the genome, including all extra-chromosomal DNA. The hybrid sequencing via Oxford Nanopore Technologies and Illumina platform (ONT-Illumina hybrid sequencing) was demonstrated to be suitable, providing complete information of the chromosome and all plasmids. However, the PacBio platform may be used for validation of the chromosome sequence, or when there are no small plasmids in the genome. After sequencing, a complete *de-novo* genome assembly should be performed to identify the locations of the AMR genes, if present in the genome.
3. **Species identification**. The 16S rRNA gene may be utilized to identify the most probable species of the unknown strain. The average nucleotide identity (ANI) based on the whole-genome sequence (≥ 95–96% to the type strain of the species)^10^ should be used to validate the result obtained.
4. **Identification of virulence and undesirable genes**. Genes responsible for virulence and undesirable properties may be identified using publicly available databases and manually inspected to confirm its identity and function. Care should be taken in the interpretation of the result as genes involved in survival and adaptation should not be considered as virulence genes for non-pathogenic bacteria. The search using the Kyoto Encyclopedia of Genes and Genomes (KEGG) database^13^ (available at https://www.kegg.jp) for the pathways and genes as outlined in Table 6 was shown to be efficient for this purpose.

**Table 6:** List of the KEGG pathways that should be inspected for virulence and undesirable genes.

Map ID	Name	Gene ID	Enzyme name	Function
**Under “Brite” Genes and Proteins; Protein families: Signaling and cellular processes**				
Ko02042	Bacterial toxins			Virulence factor
**Under “Pathway”**				
**Carbohydrate metabolism (for D-lactate formation)**				
00620	Pyruvate metabolism	K22373	lactate racemase [EC:5.1.2.1]	D-lactate < -> L-lactate
		K03778	D-lactate dehydrogenase [EC:1.1.1.28]	Pyruvate < -> D-lactate
**Lipid metabolism (for bile salt deconjugation and secondary bile acid biosynthesis)**				
00120	Primary bile acid biosynthesis	K01442	Choloylglycine hydrolase/bile salt hydrolase [EC:3.5.1.24]	Bile salt deconjugation
00121	Secondary bile acid biosynthesis		complete pathway	possible carcinogenic substances
**Amino acid metabolism (for biogenic amine formation)**				
00310	Lysine degradation	K01582	Lysine decarboxylase [EC:4.1.1.18]	production of cadaverine
		K23385	D-ornithine/D-lysine decarboxylase [EC:4.1.1.116]	production of cadaverine
00330	Arginine and proline metabolism	complete pathway for biogenic amine formation		
		K01583, K01584, K01585, K02626	Arginine decarboxylase [EC:4.1.1.19]	arginine -> agmatine
		K01480	Agmatinase [EC:3.5.3.11]	agmatine -> putrescine
		K00797	Spermidine synthase [EC:2.5.1.16]	putrescine -> spermidine, spermine
		K01476	Arginase [EC:3.5.3.1]	arginine -> ornithine
		K01581	Ornithine decarboxylase [EC:4.1.1.17]	ornithine -> putrescine
00340	Histidine metabolism	K01590	Histidine decarboxylase [EC:4.1.1.22]	histidine -> histamine
00350	Tyrosine metabolism	K22329, K22330, K01592, K18933	Tyrosine decarboxylase [EC:4.1.1.25]	tyrosine -> tyramine
00380	Tryptophan metabolism	K01593	Tryptophan decarboxylase [EC:4.1.1.28]	tryptophan -> tryptamine
5. **Identification of functional and transferrable AMR genes**. The MICs for specific antimicrobial drugs should be identified as recommended by EFSA, 2012^19^. The AMR genes, especially those responsible for the resistance phenotype, should be determined. The search using the KEGG database under “Brite ko01504: Antimicrobial resistance genes” was shown to be efficient for this purpose. If present, the genes’ location should be determined to assess their transferability. The AMR gene located in conjugative plasmids, plasmids, and intact prophages should be regarded as having a high probability of transfer. A web-based tool such as oriTfinder^28^ (available at https://bioinfo-mml.sjtu.edu.cn/oriTfinder/) may be used to identify the origin of transfer (*oriT*), the essential element for self-transmitted conjugative plasmids. Similarly, a web-based tool such as PHASTER^24,25^ (available at http://phaster.ca/) may be employed for identification of existing prophages in the genome. Strains found to posses functional and transferrable AMR genes should not be used for consumption.
6. **Identification of antimicrobial drug production capability**. For the antimicrobial drugs of concern, the latest revision of the World Health Organization’s List of Critically Important Antimicrobials for human medicine (WHO CIA list)^26^ should be used as reference. Since there is no database available for identification of the genes involved in the biosynthesis of all antimicrobials in the list, the KEGG database is currently suggested as the best resource for this purpose. The relevant pathways that should be investigated were provided in Table 7. If production of a specific antimicrobial drug is suspected or was reported for the species, but not yet included in the database, the particular genes involved in the production pathway should be manually searched, and the actual ability of the strain to produce the antimicrobial drugs should be tested.

**Table 7:** List of the KEGG pathways related to the biosynthesis of antimicrobial drugs with clinical importance.

map_ID	map_Name	Products
**Metabolism of terpenoids and polyketides**		
map01052	Type I polyketide structures	several antibiotics including erythromycin, oleandomycin, tylosin, rifamycin
map00522	Biosynthesis of 12-, 14- and 16-membered macrolides	several antibiotics including erythromycin, oleandomycin, tylosin
map01051	Biosynthesis of ansamycins	rifamycin
map00253	Tetracycline biosynthesis	tetracyclin
map01054	Nonribosomal peptide structures	several antibiotics including Bacitracin, Pristinamycin IA
map01055	Biosynthesis of vancomycin group antibiotics	vancomycin
**Biosynthesis of other secondary metabolites**		
map00311	Penicillin and cephalosporin biosynthesis	penicillin, cephalosporin
map00332	Carbapenem biosynthesis	carbapenem antibiotics
map00261	Monobactam biosynthesis	monobactam antibiotics
map00331	Clavulanic acid biosynthesis	cluvulanic acid
map00521	Streptomycin biosynthesis	streptomycin
map00524	Neomycin, kanamycin and gentamicin biosynthesis	Neomycin, kanamycin and gentamicin

In conclusion, it should be emphasised that the *in silico* analysis used in this study represents only the first step in the safety assessment of a microbial strain and cannot fully substitute the *in vivo* safety assessment and monitoring of the undesirable side effects. However, this analysis can be used to screen for high-risk strains without the need for animal testings. It also provides valuable information for identification of potential risk and specific areas that should be further investigated. The guideline proposed here can be used to facilitate the development of new and safe microbial cultures as well as to ensure public health safety.

## Methods

### Culture condition

*L. plantarum* BCC9546 was obtained from BIOTEC culture collection, Thailand. The bacterium was maintained and grown in De Man, Rogosa and Sharpe (MRS) medium (Difco Laboratories Inc., USA). The culture was grown in MRS broth at 30 °C for 12–16 h to obtain cells for DNA extraction.

### Genomic DNA extraction

The genomic DNA was extracted using Wizard Genomic DNA Purification kit (Promega Corporation, USA) according to the manufacturer’s protocol with some modifications. The modifications included addition of 25 U/mL mutanolysin (Sigma-Aldrich, USA) in the 1 mg/mL lysozyme resuspending solution, incubation at 37 °C overnight for complete cell lysis, and additional centrifugation at 10,000 × g for 3 min in the protein precipitation step. The DNA pellet was dissolved in DNase/RNase-free water. The concentration and quality of the DNA were measured using NanoDrop ND-1000 spectrophotometer (Thermo Fisher Scientific, USA). High purity genomic DNA expressing an OD_260_/OD_280_ ratio of 1.8–2.0 and an OD_260_/OD_230_ ratio of 2.0–2.2 was used for whole-genome sequencing. The integrity of the genomic DNA was visualised using 1% agarose gel electrophoresis in 0.5× TBE buffer. The gel was stained in 5 µg/mL ethidium bromide solution for 5 min and destained in tap-water for 10 min. The gel image was captured using Gel Doc XR + Imaging System (Bio-rad, USA) with Image Lab 5.1 software and setting for optimised exposure time for the intense band.

### Plasmid DNA extraction

Plasmid DNA was extracted using ZymoPURE Plasmid Miniprep kit (Zymo Research Corporation, CA, USA). The manufacturer’s protocol was modified to include the addition of 10 mg/mL lysozyme and 25 U/mL mutanolysin to the P1 buffer, and an additional incubation step at 37 °C for one h to ensure complete cell lysis. The possible number of plasmids and their size were visualised using 0.5% agarose gel electrophoresis in 0.5× TBE buffer.

### Whole-genome sequencing and genome assembly

The genome of *L. plantarum* BCC9546 was first sequenced using PacBio sequencing platform (RSII SMRT cell, Pacific Biosciences at McGill University and Génome Québec Innovation Centre, Canada). The sequencing reads were assembled *de novo* using Celera Assembler in HGAP (Hierarchical Genome-Assembly Process) workflow^29^. An additional whole-genome sequencing based on hybrid technologies was performed to obtain the full genomic information of the strain. Oxford Nanopore Technologies (ONT) (Rapid sequencing kit, MinION^TM^ device, Oxford Nanopore Technologies, UK) was used as an alternative long-read sequencing technique, and a short-read high-throughput Illumina platform (NextSeq^®^ 500 high output kit v2 (300 cycles), Illumina, Inc., USA) was used to improve the accuracy of the final sequence. The hybrid ONT-Illumina sequencing was conducted at the University of Arkansas for Medical Sciences, USA. An assembly pipeline for bacterial genomes, Unicycler, was used to assemble and polish the hybrid sequence^30^.

### Gene prediction and functional annotation

Gene prediction and computational annotation of protein-coding genes were performed using MAKER2 annotation pipeline package^31^. The prokaryotic gene sequences from NCBI database release 232 were used as the training data for the bundled GeneMark HMM within the MAKER2 package.

### Species identification

The species designation of the strain was first determined using the 16 S rRNA gene sequences. All copies of the 16S rRNA gene were extracted from the whole-genome data and checked for possible contamination using a web-based tool ContEst16S^32^ available at https://www.ezbiocloud.net/tools/contest16s. Similarities of the 16S rRNA gene to known bacterial species were searched using the nucleotide basic local alignment search tool (blastn) available at https://blast.ncbi.nlm.nih.gov. The similarity cut-off value at ≥ 98.7% was used for initial species classification using 16S rRNA sequences^9^. Then, the average nucleotide identity (ANI) to the type strains of selected species were determined using JSpecies Web Server (JSpeciesWS)^33^ available at http://jspecies.ribohost.com/jspeciesws. The ANI value of ≥95–96% was used as the criterion to confirm the species of the strain^10^.

### Determination of virulence factors and undesirable genes

The presence of virulence factors and toxin genes in the BCC9546 genome were searched using the virulence factor database (VFDB)^12^ (last updated: Jun 17, 2019) available at http://www.mgc.ac.cn/cgi-bin/VFs/v5/main.cgi. Two search criteria, i.e., a stringent search using the cut-off values at >80% identity, >60% coverage; and a less stringent search with the cut-off values at >60% similarity, >60% coverage, and E-value <1e-10 were used to identify the possible virulence genes for further investigation. In addition, the BlastKOALA search tool in the Kyoto Encyclopedia of Genes and Genomes (KEGG) database^13^ (Release 90.1) available at https://www.kegg.jp/ was used and inspected for virulence factors and undesirable genes as listed in Table 6. All genes identified from their similarities to those in the databases were manually confirmed using the protein basic local alignment search tool (blastp) suite with non-redundant protein sequences (nr) database available at https://blast.ncbi.nlm.nih.gov/ ^34,35^.

### Determination of antimicrobial resistance (AMR) properties

BCC9546 was investigated for AMR properties by both phenotypic and genotypic methods. The resistance phenotype of the strain was investigated as recommended by the European Food Safety Authority (EFSA)^19^. The strain’s susceptibility to seven antimicrobial drugs, i.e., ampicillin, gentamicin, kanamycin, erythromycin, clindamycin, tetracycline, and chloramphenicol was determined. The minimum inhibitory concentration (MIC) for each antimicrobial was evaluated through the microdilution method as described in the international standard ISO 10932:2010^20^. In brief, the strain was grown on MRS agar plate for 16–24 h. The colonies were then suspended in 5 mL sterile 0.85% NaCl solution to reach an OD_625_ of 0.16–0.2. The bacterial suspension was diluted 500 times in double-strength LSM broth (90% IST broth (ISO-sensitest broth, Oxoid Ltd., UK): 10% MRS broth). Fifty microlitres of the diluted bacterial suspension was added into wells containing 50 microliters of double-strength two-fold dilution series of the tested antimicrobials. Ampicillin, erythromycin, and kanamycin were purchased from Bio Basic Inc, USA. Chloramphenicol and clindamycin were purchased from United States Biological, USA. Gentamicin and tetracycline were purchased from AppliChem GmbH, Germany. The antimicrobial solutions were prepared by dissolving each antimicrobial powder in an appropriate solvent and adjusted for the potency as suggested in the ISO standard. *Lactobacillus paracasei* ATCC 334 and *Bifidobacterium longum* ATCC 15707 were used as quality control strains to ensure the performance of the prepared antimicrobial solutions. A well containing the test strain and the medium containing the solvent used to dissolve the antimicrobial at the highest concentration was used as the positive control, and a well containing the medium but without the test strain and the antimicrobial was used as the negative control. Since the intended use of BCC9546 is for human consumption, all incubation steps were conducted at 37 °C, under anaerobic condition (10% H_2_: 10% CO_2_: 80% N_2_). The MIC for each antimicrobial was determined, in triplicate, after incubating for 48 h.

The genetic determinants conferring AMR in the genome were searched using three publicly available databases, i.e., Comprehensive Antibiotic Resistance Database (CARD, RGI 5.0.0, CARD 3.0.3) available at https://card.mcmaster.ca/ ^21^, ResFinder (ResFinder 3.2, database 2019-07-19) available at https://cge.cbs.dtu.dk/services/ResFinder/ ^22^, and KEGG database (Release 90.1) using BlastKOALA search tool and inspected under “Brite ko01504: Antimicrobial resistance genes”^13^.

Transferability of the AMR genes found in the genome was investigated by their locations in two mobile elements, i.e., plasmids and bacteriophages. The existence of prophages in the genome was searched using PHASTER tool available at http://phaster.ca/ (Prophage/Virus DB last updated on Aug 3, 2017)^24,25^. For plasmids, the possibility for self-transmission through conjugation was investigated using oriTfinder, a web-based tool for identification of the origin of transfers in DNA sequences available at https://bioinfo-mml.sjtu.edu.cn/oriTfinder/ (database version: 1.1, May 2017)^28^.

### Determination of antimicrobial drug production capability

To assess the capability of the strain for the production of antimicrobial drugs with clinical importance, the World Health Organization’s complete list of critically important antimicrobials (WHO CIA list)^26^ was used as a reference for the antimicrobials of interest. The genome was searched and examined for completeness of the pathways involved in antimicrobial drug biosynthesis in the KEGG database as shown in Table 7.

## Supplementary information

Supplementary information.

Supplementary dataset 1.

Supplementary dataset 2.

## Data Availability

The complete genome of *L. plantarum* BCC9546 consisting of one chromosome and five plasmids were deposited in GeneBank (accession number CP044500-CP044505).

## References

[1] Cannon JP, Lee TA, Bolanos JT, Danziger LH (2005). Pathogenic relevance of *Lactobacillus*: A retrospective review of over 200 cases. Eur. J. Clin. Microbiol. Infect. Dis..

[2] Salminen MK (2006). *Lactobacillus* Bacteremia, Species Identification, and Antimicrobial Susceptibility of 85 Blood Isolates. Clin. Infect. Dis..

[3] Kayser FH (2003). Safety aspects of enterococci from the medical point of view. Int. J. Food Microbiol..

[4] Santiyanont P (2019). Dynamics of biogenic amines and bacterial communities in a Thai fermented pork product *Nham*. Food Res. Int..

[5] Valyasevi, R. *et al*. The microbiology and development of starter culture for *nham*, the traditional Thai pork sausage. in *Seventeenth International Conference of the International Committee on Food Microbiology and Hygiene (ICFMH)* 709–711 (1999).

[6] Pariza MW, Gillies KO, Kraak-Ripple SF, Leyer G, Smith AB (2015). Determining the safety of microbial cultures for consumption by humans and animals. Regul. Toxicol. Pharmacol..

[7] Surachat K, Sangket U, Deachamag P, Chotigeat W (2017). *In silico* analysis of protein toxin and bacteriocins from *Lactobacillus paracasei* SD1 genome and available online databases. PLoS One.

[8] González-Escalona, N., Allard, M. A., Brown, E. W., Sharma, S. & Hoffmann, M. Nanopore sequencing for fast determination of plasmids, phages, virulence markers, and antimicrobial resistance genes in Shiga toxin-producing *Escherichia coli*. *PLoS One* 14 (2019). 10.1371/journal.pone.0220494 PMC6667211 31361781

[9] Chun J (2018). Proposed minimal standards for the use of genome data for the taxonomy of prokaryotes. Int. J. Syst. Evol. Microbiol..

[10] Richter M, Rosselló-Móra R (2009). Shifting the genomic gold standard for the prokaryotic species definition. Proc. Natl. Acad. Sci. USA.

[11] Senan S, Prajapati JB, Joshi CG (2015). Feasibility of Genome-Wide Screening for Biosafety Assessment of Probiotics: A Case Study of *Lactobacillus helveticus* MTCC 5463. Probiotics Antimicrob. Proteins.

[12] Liu B, Zheng D, Jin Q, Chen L, Yang J (2018). VFDB 2019: a comparative pathogenomic platform with an interactive web interface. Nucleic Acids Res..

[13] Kanehisa M, Sato Y, Morishima K (2016). BlastKOALA and GhostKOALA: KEGG Tools for Functional Characterization of Genome and Metagenome Sequences. J. Mol. Biol..

[14] Zhang ZY (2012). Safety assessment of *Lactobacillus plantarum* JDM1 based on the complete genome. Int. J. Food Microbiol..

[15] EFSA Panel on Biological Hazards (BIOHAZ). (2011). Scientific Opinion on risk based control of biogenic amine formation in fermented foods. EFSA J..

[16] Bianchetti DGAM (2018). D-lactic acidosis in humans: systematic literature review. Pediatr. Nephrol..

[17] Joint FAO/WHO Working Group. Report on Drafting Guidelines for the Evaluation of Probiotics in Food. London, Ontario, Canada (2002)

[18] Begley M, Hill C, Gahan CGM (2006). Bile salt hydrolase activity in probiotics. Appl. Environ. Microbiol..

[19] EFSA Panel on Additives and Products or Substances used in Animal Feed (FEEDAP). (2012). Guidance on the assessment of bacterial susceptibility to antimicrobials of human and veterinary importance. EFSA J..

[20] ISO 10932:2010(en). Milk and milk products - Determination of the minimal inhibitory concentration (MIC) of antibiotics applicable to bifidobacteria and non-enterococcal lactic acid bacteria (LAB). *International Organization for Standardization* (2010).

[21] Jia B (2017). CARD 2017: expansion and model-centric curation of the comprehensive antibiotic resistance database. Nucleic Acids Res..

[22] Zankari E (2012). Identification of acquired antimicrobial resistance genes. J. Antimicrob. Chemother..

[23] Philippon A, Slama P, Dény P, Labia R (2016). A structure-based classification of class A β-Lactamases, a broadly diverse family of enzymes. Clin. Microbiol. Rev..

[24] Zhou Y, Liang Y, Lynch KH, Dennis JJ, Wishart DS (2011). PHAST: A Fast Phage Search Tool. Nucleic Acids Res..

[25] Arndt D (2016). PHASTER: a better, faster version of the PHAST phage search tool. Nucleic Acids Res..

[26] World Health Organization (WHO). Critically important antimicrobials for human medicine, 6^th^ revision. https://www.who.int/foodsafety/publications/antimicrobials-sixth/en/ (2019).

[27] Hong HA (2008). The safety of *Bacillus subtilis* and *Bacillus indicus* as food probiotics. J. Appl. Microbiol..

[28] Li X (2018). OriTfinder: A web-based tool for the identification of origin of transfers in DNA sequences of bacterial mobile genetic elements. Nucleic Acids Res..

[29] Chin CS (2013). Nonhybrid, finished microbial genome assemblies from long-read SMRT sequencing data. Nat. Methods.

[30] Wick RR, Judd LM, Gorrie CL, Holt KE (2017). Unicycler: Resolving bacterial genome assemblies from short and long sequencing reads. PLoS Comput. Biol..

[31] Holt C, Yandell M (2011). MAKER2: An annotation pipeline and genome-database management tool for second-generation genome projects. BMC Bioinformatics.

[32] Lee I (2017). ContEst16S: An algorithm that identifies contaminated prokaryotic genomes using 16S RNA gene sequences. Int. J. Syst. Evol. Microbiol..

[33] Richter M, Rosselló-Móra R, Oliver Glöckner F, Peplies J (2016). JSpeciesWS: A web server for prokaryotic species circumscription based on pairwise genome comparison. Bioinformatics.

[34] Altschul SF (2005). Protein database searches using compositionally adjusted substitution matrices. FEBS J..

[35] Altschul SF (1997). Gapped BLAST and PSI-BLAST: A new generation of protein database search programs. Nucleic Acids Res..

