# Supplementary material for: Safety Assessment of a Nham Starter Culture Lactobacillus plantarum BCC9546 via Whole-genome Analysis
Source: Sci Rep. 2020 Jun 24;10:10241. doi: 10.1038/s41598-020-66857-2 (PMC7314741; doi:10.1038/s41598-020-66857-2)
Supplement: Supplementary file 1 — Supplementary information. [file 41598_2020_66857_MOESM1_ESM.docx]

**Supplementary information**

**Safety Assessment of a *Nham* Starter Culture *Lactobacillus plantarum* BCC9546 via Whole Genome Analysis**

Nipa Chokesajjawatee^1*^, Pannita Santiyanont^1^, Kanittha Chantarasakha^1^, Kanokarn Kocharin^1^, Chinae Thammarongtham^1^, Supatcha Lertampaiporn^1^, Tayvich Vorapreeda^1^, Tanawut Srisuk^2^, Thidathip Wongsurawat^3^, Piroon Jenjaroenpun^3^, Intawat Nookaew^3^, Wonnop Visessanguan^1^

**D-lactic acid production**

**Method:** Determination of D-lactic acid production.

The bacterial strain BCC9546 was grown in De Man, Rogosa and Sharpe (MRS) (Becton Dickinson and Company, USA) broth at 37 °C for 18 h. The cell-free supernatant was subjected to D- and L- lactic acid analysis using Dionex HPLC system equipped with UV detector at 230 nm, chiral column (Shodex CRX853), using 0.5 mM CuSO_4_ as mobile phase at a flow rate of 0.25 ml/min at 40 °C. The optical purity of lactic acid is defined as follows: optical purity (%) = |D-lactic acid concentration − L-lactic acid concentration|/(D-lactic acid concentration + L-lactic acid concentration) × 100.

**Results:** The strain BCC9546 produced D-lactic acid with ca. 50% optical purity

| Sample name | L-Lactic  (g/L) | D-Lactic  (g/L) | DL-Lactic acid (g/L) | optical purity (%) |
| --- | --- | --- | --- | --- |
| LP9546_1 | 5.66 | 16.38 | 22.03 | 48.65 |
| LP9546_2 | 5.36 | 16.33 | 21.69 | 50.53 |

**Genomic and Plasmid DNA extraction**

**Results:** Genomic and Plasmid DNA extraction

**Figure S1.** The uncropped, full-length agarose gel electrophoresis of DNA extracts from BCC9546. **(a)** Genomic DNA running on 1% agarose gel: M, 250 ng DNA size marker (GeneRuler DNA Ladder Mix, Thermo Scientific); 1-2, 250 ng BCC9546 genomic DNA extract. **(b)** Plasmid DNA running on 0.5% agarose gel: Ms, 250 ng supercoiled DNA Ladder (New England BioLabs); p1, 50 ng BCC9546 plasmid DNA extract; p2, 120 ng BCC9546 plasmid DNA extract.

**Results: Hemolysis on sheep blood agar**


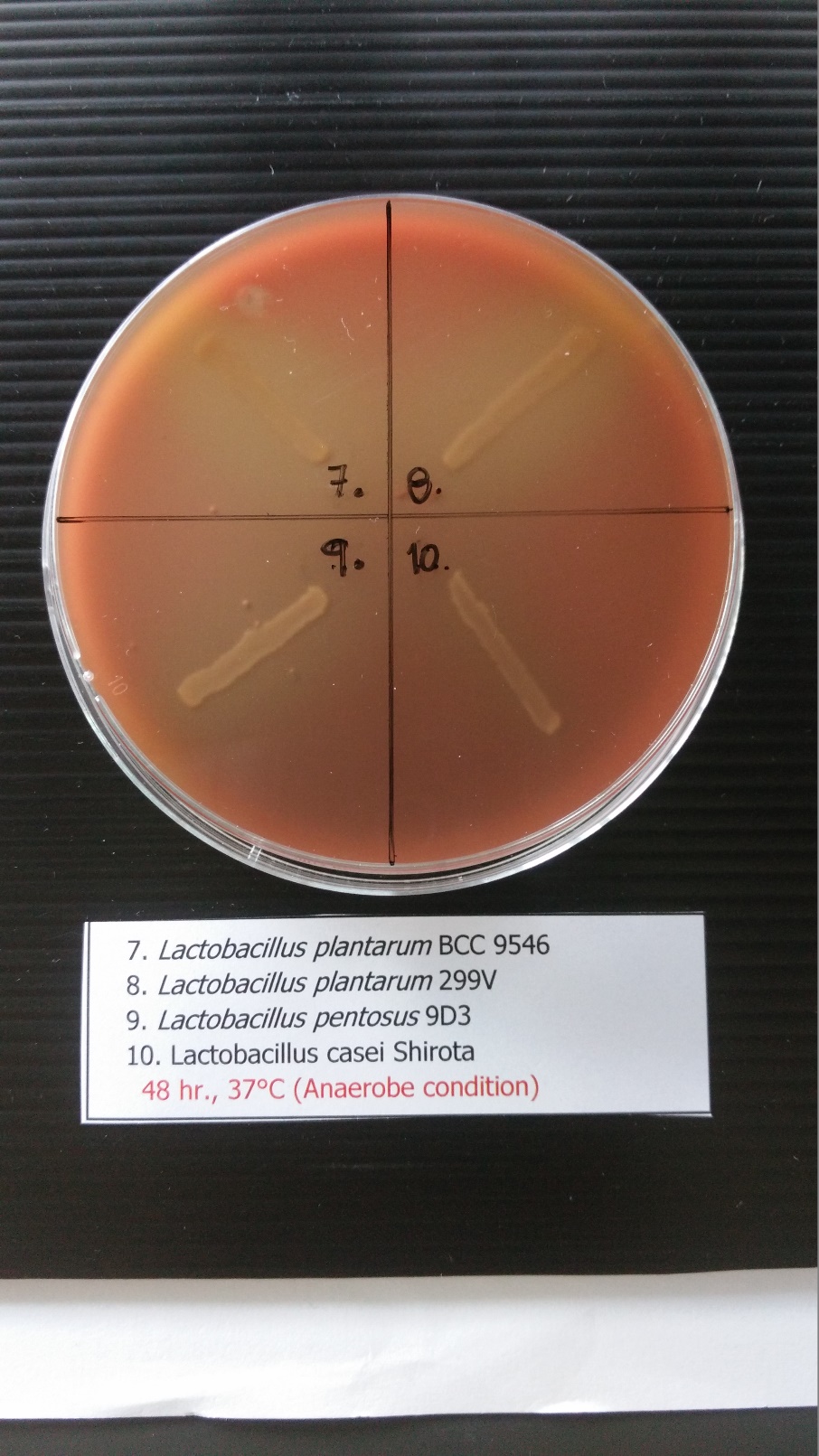


**Figure S2.** Zone of hemolysis on sheep blood agar of *L. plantarum* BCC9546 (7) and *L. plantarum* 299V (8), after incubation at 37 °C for 48 h in anaerobic condition.
